# Epigenetics, Nervous System Tumors, and Cancer Stem Cells

**DOI:** 10.3390/cancers3033525

**Published:** 2011-09-13

**Authors:** Irfan A. Qureshi, Mark F. Mehler

**Affiliations:** 1 Rosyln and Leslie Goldstein Laboratory for Stem Cell Biology and Regenerative Medicine, Albert Einstein College of Medicine, Bronx, New York, NY 10461, USA; E-Mail: irfan@jhu.edu; 2 Institute for Brain Disorders and Neural Regeneration, Albert Einstein College of Medicine, Bronx, New York, NY 10461, USA; 3 Department of Neurology, Albert Einstein College of Medicine, Bronx, New York, NY 10461, USA; 4 Department of Neuroscience, Albert Einstein College of Medicine, Bronx, New York, NY 10461, USA; 5 Department of Psychiatry and Behavioral Sciences, Albert Einstein College of Medicine, Bronx, New York, NY 10461, USA; 6 Rose F. Kennedy Center for Research on Intellectual and Developmental Disabilities, Albert Einstein College of Medicine, Bronx, New York, NY 10461, USA

**Keywords:** cancer, cancer stem cell, nervous system tumor, chromatin, CoREST, epigenetic, glioblastoma multiforme, non-coding RNA, REST

## Abstract

Recent advances have begun to elucidate how epigenetic regulatory mechanisms are responsible for establishing and maintaining cell identity during development and adult life and how the disruption of these processes is, not surprisingly, one of the hallmarks of cancer. In this review, we describe the major epigenetic mechanisms (*i.e.*, DNA methylation, histone and chromatin modification, non-coding RNA deployment, RNA editing, and nuclear reorganization) and discuss the broad spectrum of epigenetic alterations that have been uncovered in pediatric and adult nervous system tumors. We also highlight emerging evidence that suggests epigenetic deregulation is a characteristic feature of so-called cancer stem cells (CSCs), which are thought to be present in a range of nervous system tumors and responsible for tumor maintenance, progression, treatment resistance, and recurrence. We believe that better understanding how epigenetic mechanisms operate in neural cells and identifying the etiologies and consequences of epigenetic deregulation in tumor cells and CSCs, in particular, are likely to promote the development of enhanced molecular diagnostics and more targeted and effective therapeutic agents for treating recalcitrant nervous system tumors.

## 1. Introduction

Epigenetic mechanisms are deployed in response to complex profiles of cell-extrinsic, cell-cell, and cell-intrinsic signals and are responsible for the establishment, maintenance, and refinement of cell identity and function that occurs during development and adult life [1]. Indeed, an increasing number of studies has begun to elucidate how DNA methylation, histone and chromatin modification, non-coding RNAs (ncRNAs), RNA editing, and nuclear reorganization (Table 1) orchestrate stem cell self-renewal and maintenance, lineage restriction, lineage commitment, cell fate specification, progressive stages of maturation, and terminal differentiation. These regulatory mechanisms seem to promote the formation of relatively “open” and “poised” epigenetic states permissive for transcriptional activity that are associated with multi-lineage potential in stem and progenitor cells and to mediate the execution of lineage-specific gene expression programs, including the silencing of genes associated with alternate cell fates, in more mature cellular species.

Not surprisingly, epigenetic deregulation seems to be one of the hallmarks of cellular transformation, and our emerging understanding of the roles played by epigenetic factors in cancer biology has led to a number of recent advances in clinical oncology [2]. For example, diagnostic and prognostic tests that detect mutations in epigenetic factors and disease-associated variations in DNA methylation and ncRNA expression profiles have reached the marketplace, and drugs that target DNA methylation and histone modification pathways have been approved by the Food and Drug Administration for selected indications. A broad range of related, as well as novel, epigenetic diagnostic and treatment strategies are also being developed very actively and are expected to revolutionize cancer care.

In this review, we briefly describe the major epigenetic mechanisms and highlight their specific roles in regulating neural cell identity and function. We also illustrate how epigenetic deregulation, and the corresponding loss of normal neural cell identity and function, seems be a cardinal feature of pediatric and adult nervous system tumors including glioblastoma multiforme (GBM), medulloblastoma and neuroblastoma—which are amongst the most challenging cancers to treat because they exhibit considerable resistance to standard therapies and high recurrence rates. The cancer stem cell (CSC) hypothesis proposes that tumor maintenance, progression, treatment resistance, and recurrence are principally driven by a small population of stem–like cells present within a tumor. As such, we also draw attention to emerging evidence of epigenetic deregulation in tumor initiating and propagating cells derived from nervous system tumors—cells that are functionally defined as CSCs. Further, we consider the potential impact of epigenetic processes in promoting the establishment and maintenance of the CSC state, including the mediation of the bidirectional communications that exist between CSCs and the tumor microenvironment.

## 2. Epigenetic Mechanisms and Their Roles in Establishing and Maintaining Neural Cell Identity

### 2.1. DNA Methylation

DNA methylation refers to methylation of cytosine residues in genomic DNA, which leads to the formation 5-methycytosine (5mC) [1]. This covalently modified nucleotide is found prominently in gene regulatory regions (e.g., promoter elements) and in other regions (e.g., inter- and intra-genic sequences and repetitive elements). Members of the DNA methyltransferase (DNMT) family of enzymes are responsible for promoting *de novo* methylation and for maintaining methylation. Factors implicated in DNA demethylation have more recently been identified and include DNA excision repair, cytidine deaminase, and Gadd45 proteins. Methyl-CpG-binding domain (MBD) proteins bind specifically to methylated DNA and recruit additional regulatory factors to these loci. This DNA methylation machinery and the corresponding profiles of DNA methylation are dynamically regulated during development, adult life, and aging in a tissue- and cell type-specific manner that is environmentally sensitive (and activity-dependent in the nervous system). DNA methylation is, in concert with other epigenetic mechanisms, involved in mediating a broad range of cellular processes including transcriptional repression (and rarely activation) at individual gene loci and more globally; X chromosome inactivation (XCI) and genomic imprinting; and maintenance of genomic integrity.

DNA methylation plays a role in establishing and maintaining neural cell identity [1]. For example, one recent study showed that in a cellular differentiation paradigm progressing from embryonic stem cells (ESCs) to lineage-committed neural progenitor cells (NPCs), hundreds of promoter regions are methylated in NPCs, including those associated with pluripotency and germline-specific genes, suggesting that DNA methylation is responsible for silencing these genes and promoting neural lineage commitment [3]. Another study showed that, in postnatal neural stem cells (NSCs), DNMT3A methylates intergenic regions and gene bodies flanking proximal promoters regions of a large number of genes, including many regulators of neurogenesis, DNMT3A-dependent non-proximal promoter methylation promotes the expression of these neurogenic genes, and DNMT3A is required for neurogenesis [4]. These observations suggest that DNA methylation mediates neural lineage elaboration by silencing genes associated with pluripotency and activating neuronal lineage-specific genes.

Another modified cytosine residue, 5-hydroxymethylcytosine (5hmC) is also implicated in the epigenetic regulation of cell identity, though its functional roles are not as well understood. 5hmC is likely generated by the oxidation of 5mC, which is catalyzed by the Ten-Eleven Translocation (TET) family of enzymes that are highly expressed in ESCs [5,6]. Mechanistically, 5hmC inhibits the binding of MBD proteins to DNA implying that its role may, in part, be to counterbalance that of 5mC [7,8]. In fact, recent analyses of 5hmC (and TET proteins) in ESCs and induced pluripotent cells suggest that 5hmC is associated with actively transcribed genes in pluripotent cells and, further, that the balance between 5hmC and 5mC levels is important for regulating the switch from a pluripotent to a lineage committed cellular species [6,9,10]. Additional studies have found that 5hmC is widely distributed in brain and is particularly abundant in regions involved in higher cognitive functions, such as the cortex and hippocampus [11-13]. Interestingly, characterizing the distribution of 5hmC in DNA from human brain frontal lobe tissue reveals that 5hmC is selectively targeted to promoter elements and gene bodies rather than intergenic regions and, further, that genes associated with these regions are significantly enriched for those involved in neural development and patterning [13]. These findings suggest that the cell-type specific regulation of this epigenetic modification, like that of 5mC, also plays a role in the establishment and maintenance of neural cell identity.

### 2.2. Histone and Chromatin Modifications

Chromatin refers to the packaging of genomic DNA, along with histone and non-histone proteins and associated factors, within the cell nucleus [1]. The nucleosome is the most essential unit of chromatin. It is formed by 147 base pairs of DNA wrapped around an octamer comprised of two of each of the classical histone proteins (*i.e.*, H2A, H2B, H3, H4) and by linker DNA and histones (*i.e.*, H1). A series of nucleosomes forms the characteristic “beads-on-a-string” configuration that can, in turn, fold into secondary and tertiary structures representing varying degrees of condensation. For example, higher order chromatin conformations include loosely packaged euchromatin and more densely packaged heterochromatin. Chromatin states can be modulated at multiple levels within this hierarchy. Classical histone proteins can be replaced by variant histones (e.g., H2A.Z), with distinct properties. Specific classes of enzymes are responsible for regulating reversible histone post-translational modification levels including acetylation (*i.e.*, histone acetylases and deacetylases [HATs and HDACs]) as well as methylation, phosphorylation, SUMOylation, ADP-ribosylation and others. Additional enzymes and macromolecular complexes promote nucleosome and higher order chromatin (e.g., Polycomb Group [PcG] and Trithorax Group [TrxG] proteins) remodeling. Histone-, nucleosome-, and higher order chromatin-modifying enzymes may act alone; or, because strata within this chromatin hierarchy are interconnected, these factors may be integrated into highly modular complexes, such as the REST and the functionally related CoREST epigenetic regulatory complexes. Rearrangement of chromatin is responsible for the establishment of more or less compacted configurations that play dynamic regulatory, structural, and other functional roles within the cell. These effects can include modulating interactions between DNA sequences with each other and with diverse nuclear factors including ncRNAs, DNA binding proteins, transcriptional co-regulators, transcription factors, and additional nuclear machineries, such as those involved in mediating the processes of transcription and DNA replication and repair. Like DNA methylation, histone and chromatin modifications are involved in mediating a broad range of cellular processes including transcriptional repression and activation at individual gene loci and more globally; XCI and genomic imprinting; and maintenance of genomic integrity. Histone and chromatin modifying factors and the corresponding profiles of chromatin modifications are, like DNA methylation, dynamically regulated during development, adult life, and aging in a tissue- and cell type-specific manner that is environmentally sensitive.

Histone modifications and chromatin remodeling play a role in establishing and maintaining neural cell identity (reviewed in [14,15]). For example, we recently demonstrated that developmental stage-specific deployment of REST and CoREST complexes is responsible for controlling NSC-mediated neural lineage elaboration including neuronal and glial subtype specification, progressive maturation, and terminal differentiation (*i.e.*, oligodendrocyte [OL] myelination) through dynamic and context-specific activation and repression of cell type- and maturational stage-specific gene networks [16-18]. Similarly, recent studies have shown that the histone demethylase, LSD1, regulates the proliferation of NSCs [19]; the TrxG protein, Mll1, is essential for postnatal NSC-mediated neurogenesis [5]; and, PcG proteins play a role in the temporal regulation of NPC fate by modulating neuronal to glial fate switching [20]. Moreover, another study profiled PcG-mediated histone H3 lysine (K) 27 trimethylation (H3K27me3) in ESCs, lineage committed NPCs, and terminally differentiated neurons and found dynamic changes in these marks in promoter regions of many genes including, specifically, loss of H3K27me3 from neuron-specific genes activated upon terminal differentiation in the transition from NPCs to terminally differentiated neurons [3]. Interestingly, this study also reported that promoter regions with H3K27me3 in stem cells are often associated with DNA methylation events during differentiation, highlighting how these developmental processes are likely coordinated by crosstalk between different epigenetic regulatory mechanisms.

### 2.3. Non-Coding RNAs

The great majority of human genomic DNA does not code for protein; non-coding sequences are nevertheless transcribed, forming various classes of ncRNAs. These ncRNAs are more abundant than protein-coding RNAs in human cells [21]. Certain classes of ncRNAs, such as transfer RNAs and ribosomal RNAs, are well known. Other classes of ncRNAs have more recently been recognized and novel classes are being identified at a rapid pace. These include short ncRNAs such as endogenous short-interfering RNAs (siRNAs), microRNAs (miRNAs), PIWI-interacting RNAs and small nucleolar RNAs (snoRNAs), and long ncRNAs (lncRNAs) such as long intergenic ncRNAs (lincRNAs). NcRNAs have expression profiles that are cell-, tissue-, and developmental stage-specific (and activity-dependent in the nervous system) [22]. NcRNAs have diverse regulatory, structural, and other operative roles, including many that are still emerging [23]. Known molecular functions for ncRNAs include mediating and/or modulating aspects of DNA methylation, chromatin modification, transcription, and RNA post-transcriptional processing, transport, and translation [24].

MiRNAs, the best-characterized class of ncRNAs, regulate target messenger RNAs (mRNAs) through post-transcriptional mechanisms [25]. Individual miRNAs have the potential to modulate hundreds of complementary mRNA molecules through complete or partial sequence-specific interactions. Single-stranded miRNA molecules bind to mRNAs primarily in 3′ untranslated regions (UTRs) as well as in certain coding regions and 5′ UTRs, preventing the translation of these mRNAs or sequestering them for storage or degradation via the RNA-induced silencing complex. Conversely, a single mRNA may be the target of multiple distinct miRNAs.

The functions of lncRNAs, the most abundant class of ncRNAs, are not as well understood. Only a few lncRNAs have been studied in detail, such as the *X inactivation-specific transcript*, which mediates X chromosome inactivation. More recently, however, lncRNA biogenesis and function have been examined more systematically. LncRNAs are defined as transcripts longer than 200 nucleotides that can be, but are not necessarily, 5′ capped, polyadenylated, and spliced like mRNAs [26-29]. Some lncRNAs are derived from intergenic regions, whereas others are transcribed in antisense, bi-directional, or overlapping orientations relative to protein-coding genes—genes that may be specifically regulated by their associated lncRNAs. LncRNAs can form a range of functional secondary structures, including particular protein interaction domains [30-32]. LncRNAs can be cleaved into shorter ncRNAs (e.g., siRNAs, snoRNAs and miRNAs) [33]. Thus, lncRNAs represent highly versatile molecules. Correspondingly, these factors have a broad spectrum of functions, including the ability to recruit transcriptional and epigenetic (e.g., chromatin remodeling complexes) regulatory factors to specific genomic sites, to form of nuclear subdomains (e.g., paraspeckles), to mediate nuclear-cytoplasmic transport of proteins, and to control translation (e.g., local protein synthesis in synaptic compartments) [34,35]. Moreover, lncRNAs are implicated in mediating a wide range of normal and pathological processes, from development, plasticity, and homeostasis to cancer metastasis [36].

Many studies have begun to elucidate the expression and function of miRNAs in NSC self-renewal, maintenance and lineage restriction, neuronal and glial lineage commitment, neuronal and glial subtype specification, progressive maturation, and plasticity [37]. For example, one important study showed that *miR-124*, the most abundant miRNA in the central nervous system (CNS), regulates the progression of NSCs into neurons [38]. Specifically, in NSCs, knocking down *miR-124* derepresses its target, Sox9, and results in disruption of neuronal lineage elaboration with arrest in the proliferative precursor state. Two recent studies demonstrated that miRNAs also regulate OL development [39,40]. Both studies showed that eliminating the presence of mature miRNAs via targeted disruption of the miRNA biogenesis factor, Dicer1, leads to profound impairments in the progressive maturation of specified OL lineage species. Further, the introduction of specific miRNAs (e.g., *miR-219* and *miR-338*) rescues the abnormal OL developmental phenotypes by modulating the expression of proteins with important roles in regulating the fidelity of OL maturation.

In addition, we, and others, have shown that lncRNAs play a role in establishing and maintaining neural cell identity. For example, one key study showed that lncRNAs are specifically expressed in the developing brain and preferentially associated with protein-coding genes expressed in the brain or involved in transcriptional regulation of CNS development. Moreover, we found 169 lncRNAs that are significantly differentially expressed during neuronal-glial fate specification and OL lineage maturation including those associated with key neural developmental genes, such as *AK053922*, a lncRNA transcribed from the *Gli3* locus, and *Sox8OT*, a lncRNA transcribed from the *Sox8* locus [41]. Others have similarly identified lncRNAs with roles in OL lineage elaboration including *Nkx2.2AS*, which is transcribed in an antisense orientation relative to Nkx2.2 [42]. Another study found more than 1,000 lincRNAs in various cell types, including neural precursor cells and identified a “brain cluster” of lncRNAs associated with hippocampal development, OL maturation, and GABAergic neuronal function [43].

Intriguingly, REST and CoREST regulate the expression levels of particular ncRNAs (*i.e.*, miRNAs and lncRNAs) [32]. In turn, specific ncRNAs can modulate the expression levels of REST and CoREST [32]. Moreover, lncRNAs are implicated in mediating the function of these and other chromatin remodeling complexes, by recruiting them to their genomic sites of actions. These observations, once again, highlight the complex crosstalk that occurs between different epigenetic regulatory mechanisms that modulate neural cell fate decisions.

### 2.4. RNA Editing

Editing is a key mechanism for generating diversity in the information content of ncRNA molecules (as well as protein-coding RNAs and even DNA) [44,45]. RNA editing refers to nucleoside modifications, including adenosine-to-inosine (A-to-I) and cytidine-to-uridine (C-to-U) deaminations in RNA molecules, catalyzed by the adenosine deaminase that act on RNA (ADAR) and the apolipoprotein B editing catalytic subunit (APOBEC) families of enzymes, respectively. ADARs are differentially expressed during development with ADAR1 and 2 preferentially found in the CNS and ADAR3 restricted to brain [46,47]. The targets of A-to-I RNA editing were initially thought to encompass protein-coding transcripts, particularly those involved in the establishment and maintenance of neuronal cell identity and synaptic transmission such as voltage-gated ion channels, ligand-gated receptors, and signal transduction molecules [44,48]. More recent studies have recognized that A-to-I RNA editing occurs primarily in ncRNA transcripts derived from *Alu* elements and in UTRs of protein-coding transcripts [49-52] and that these editing events occur at significant levels within the human brain [53].

RNA editing plays a role in establishing and maintaining neural cell identity. For example, one study showed that human ESC differentiation is generally associated with an ADAR1-mediated global decrease in the editing levels of *Alu* elements, and these changes are particularly significant during neural lineage restriction [54]. Further, knocking down ADAR1 in ESCs leads to the deregulation of various genes including, particularly, those that are involved in neuronal development. Another recent study found that, as human NPCs differentiate into neuronal and glial lineages, the levels of ADAR2 and alpha-amino-3-hydroxy-5-methyl-4-isoxazolepropionic acid (AMPA) receptor GluR2 subunit pre-mRNA editing increase [55]. This change in editing levels is associated with a transition from AMPA receptor calcium-permeability in NPCs to impermeability in more mature cells. In turn, overexpression of ADAR2 impairs NPC differentiation.

### 2.5. Nuclear Organization

Nuclear organization refers to the three dimensional arrangement of the genome, from entire chromosomes to individual gene loci, within the context of the nuclear envelope and matrix and other factors. The execution of cellular programs including DNA replication and repair, gene transcription, and RNA post-transcriptional processing and transport is associated with dynamic nuclear reorganization such as the formation and disassembly of functional nuclear domains [1,24,56-60]. The genome can undergo controlled local and long-range movements, more global reorganization, as well as other shifts (e.g., chromatin states) that have functional consequences for nuclear processes. Further, the nucleus contains a number of specialized sub-organelles comprised of particular proteins and RNA species, including some whose roles have been characterized and others that are less well understood. These heterogeneous nuclear domains can dynamically assemble and associate with specific genomic loci, interact with chromatin, move, and undergo other changes. These three-dimensional features of nuclear structure and function do not arise randomly. Rather, they are associated with particular physiological cues and cellular processes, such as cell-cycle progression and differentiation. Although the regulation and function of many nuclear features has not yet been elucidated, recent advances have highlighted the interconnected nature of nuclear organization and dynamics with epigenetic regulation, in general and in the CNS [61], both in health and in disease [62].

Nuclear reorganization plays a role in establishing and maintaining neural cell identity. For example, one important study showed how the expression of *Mash1*, a pro-neural gene, is mediated by the repositioning of the *Mash1* locus from the nuclear periphery to the interior of the nucleus, which occurs during neural lineage commitment of ESCs [63]. At the nuclear periphery, the *Mash1* locus is associated with profiles of histone modifications indicative of transcriptional repression, whereas in the interior of the nucleus, it is associated with profiles of histone modifications linked to transcriptional activation. By contrast, another study demonstrated that the expression of *PLP*, a myelin gene, is up regulated during progressive stages of OL lineage maturation, while the *PLP* locus remains at the nuclear periphery [64]. Interestingly, the increased transcription of *PLP* is associated with the local assembly of SC35 domains (a.k.a., nuclear speckles) proximal to the *PLP* gene locus. These nuclear domains are enriched in *NEAT1/2*, lncRNAs that we showed are upregulated during the progressive maturation of OLs [28], and are implicated in modulating transcription and RNA processing. Other ncRNAs have been linked unequivocally to gene regulation, nuclear organization, and OL development. The miRNA, *miR-23*, negatively regulates the expression of lamin B1 (LMNB1), a nuclear lamina protein with diverse roles in epigenetic phenomena including chromatin formation and euchromatin and heterochromatin transitions. The *LMN1B* gene is duplicated in adult-onset autosomal dominant leukodystrophy resulting in increased levels of LMNB1 that lead to defects in OL maturation and myelination accompanied by abnormalities of the nuclear envelope and myelin gene expression and localization [65,66].

## 3. Epigenetic Mechanisms and Their Roles in Cancer

### 3.1. Epigenetic Deregulation in Cancer

Along with inherited and acquired genetic abnormalities, epigenetic mechanisms are also implicated in promoting cellular transformation via mechanisms that include silencing tumor suppressor genes and activating oncogenes; deregulation of genes involved in cell growth and proliferation, stress responses, and survival and apoptosis; loss of genomic imprinting, derepression of parasitic repetitive elements, and producing genomic instability.

Indeed, epigenetic profiling in cancer has revealed a spectrum of abnormalities. Cancer cells exhibit changes in normal patterns of DNA methylation including global hypomethylation in intergenic and heterochromatic regions, which may lead to genomic instability, reactivation of transposable elements, and loss of imprinting, and local changes in regulatory regions that can modulate specific genes, including the repression of tumor suppressor genes and the activation of oncogenes. Further, hypermethylation may lead to long term gene silencing of tumor suppressor genes by promoting the establishment of “locked” repressive chromatin states. Cancer cells also exhibit abnormal profiles of histone, nucleosome, and chromatin regulation. Like changes in DNA methylation, these patterns can be deregulated globally or more locally. In fact, global H3K4me2 and H3K18ac patterns correlate with tumor phenotype and predict prognosis, treatment response, and outcome for different cancers [67-69]. By contrast, combinations of histone modifications at particular gene loci, such as repressive PcG-mediated H3K27me3 and H3K9me2 at tumor suppressor genes, are associated with deregulation of these specific genes [70]. Various cancers are further associated with mutations and other alterations in the expression and function of epigenetic factors including, for example, DNMTs, histone and chromatin modifying enzymes, as well as ncRNAs.

Cancer cells have deregulated miRNA expression profiles that are implicated in initiating and maintaining the abnormal cellular phenotype. These miRNAs target a broad range of mRNAs including those that control cell growth and proliferation, stress responses, and survival and apoptosis as well as those encoding factors involved in reading and writing the epigenetic modifications. Cancer cells also display changes in the methylation and chromatin states associated with miRNA genes. In turn, these abnormalities can lead to deregulation of miRNAs that act as tumor suppressor genes and oncogenes through effects on their target mRNAs and those that have more global effects on genomic stability [71]. Other ncRNAs, such as lncRNAs, may similarly have roles in the molecular mechanisms underlying cancer. For example, a number of lncRNAs are associated with the establishment of cell identity, including pluripotency [72-74] and fate restriction, lineage specification and maturation; with p53-mediated cell stress responses, survival, and apoptosis [75,76]; with genomic imprinting; XCI; and with cancer metastasis [36]. Cumulatively, these signatures highlight the emerging roles played by epigenetic deregulation in promoting the cancer phenotype.

### 3.2. Epigenetic Deregulation in Nervous System Tumors

#### 3.2.1. Glioblastoma Multiforme and Other Gliomas

Recent studies have revealed that GBMs exhibit complex profiles of mutations in epigenetic factors and alterations in epigenetic regulation, including those which are potentially clinically relevant [77]. For example, in depth sequencing analyses of GBMs have found mutations in many genes that encode factors with roles in epigenetic regulation, including histone deacetylases, HDAC2 and HDAC9; histone demethylases, JMJD1A and JMJD1B; histone methyltransferases, SET7, SETD7, MLL, MLL4; and methyl-CpG binding domain protein, MBD1 [78]. In addition, GBMs display deregulation of epigenetic factors, including significant over expression of particular DNMTs (e.g., DNMT3B) associated with promoter hypomethylation and differential profiles of histone modifications (*i.e.*, H3 and H4 acetylation and H3K4me2) [79]. Moreover, GBMs have down regulation of particular HDACs (e.g., class II and IV) [80] and increased copy numbers of the gene encoding BMI1, a PcG protein that regulates histone H3K27 methylation [81]. Further, the methylation status of the *O (6)-methylguanine-DNA methyltransferase (MGMT)* gene promoter is a biomarker for a GBM molecular subtype that predicts response to temozolomide and patient survival [82,83]. Interestingly, some studies profiling DNA methylation status of particular genes in GBMs and in other gliomas reveal preferential hypermethylation of specific loci in lower-grade tumors compared to higher-grade forms [84-86]. By contrast, a recent high throughput analysis demonstrated that DNA methylation remains largely stable during glioma evolution, implying that epigenetic deregulation is an early event during glioma pathogenesis [87]. These differing results provide evidence for the presence of heterogeneous epigenetic deregulation in these tumors. In fact, a detailed genomic and epigenomic evaluation of primary and secondary GBMs and other gliomas suggests that DNA methylation profiles correlate with the histology and genotype of these tumors (*i.e.*, presence of *IDH1* mutations) and can be utilized to distinguish between primary and secondary GBMs [87]. A number of studies have also identified an increasing array of miRNAs that are deregulated in GBMs. For example, a miRNA that acts as a tumor suppressor, *miR-124a*, is often down regulated in GBM promoting migration and invasion, and the degree of *miR-124a* down regulation correlates with shorter survival, clinically [88]. By contrast, a miRNA that acts as an oncogene (a.k.a., an oncomir), *miR-21*, is over expressed in GBMs, and its effects are mediated by targeting networks of tumor suppressors, including components of the p53, transforming growth factor-β, and mitochondrial apoptosis pathways [89]. Similarly, many other miRNAs are differentially expressed in GBMs and are implicated in modulating core regulatory pathways, such as those that mediate cell proliferation, apoptosis, cell cycle regulation, invasion, and angiogenesis [90]. Other ncRNAs, such as lncRNAs, may also play a role in GBMs. For example, *anti-NOS2A* is a lncRNA expressed in GBMs that is implicated in regulating the *NOS2A* gene [91]. In turn, *NOS2A* is induced in human brain tumors including GBMs and influences the efficacy of particular chemotherapeutic agents [92]. Many other genes with roles in GBMs, including oncogenes and tumor suppressor genes, are also associated with antisense lncRNAs that may regulate their related genes [93]. The expression of RNA editing enzymes and associated levels of RNA editing are also deregulated in GBMs. Specifically, ADAR1-3 are all down regulated in brain tumors, including GBM, and further, ADAR3 levels correlate with the grade of malignancy of the tumor, with a 99% decrease in ADAR3 levels found in GBMs [94]. Correspondingly, GBMs display significant global hypoediting in *Alu* element derived RNAs and gene-specific editing profiles in tumors [94]. For example, the AMPA receptor, GluR2, is hypoedited in GBM [95]. In addition, GBMs can also participate in intercellular epigenetic signaling. For example, tumor cells secrete exosomes (a.k.a., microvesicles) containing various ncRNAs *(i.e.*, miRNAs), DNA sequences, and other factors reflective of the tumor state can be delivered to healthy recipient cells throughout the body [96]. A recent analysis of the contents of exosomes derived from GBMs has revealed the presence of high levels of retrotransposon-associated RNA sequences including *Alu* and *HERV-H* retrotransposon elements, suggesting that these factors may promote tumor progression and maintenance [97].

Like other gliomas, oligodendroglial tumors also exhibit complex profiles of epigenetic deregulation that are common and likely to be related to the development and prognosis of these tumors. For example, one study showed that 74% of tumors exhibited some degree of hypermethylation associated with gene loci previously linked to the pathogenesis of oligodendroglial tumors, as well as in candidate tumor suppressor genes not previously reported to be mutated in these tumors [98]. Notably, hypermethylation of *MGMT* was significantly correlated with loss of chromosome 19q and the combined loss of chromosomes 1p and 19q [98]. These epigenetic abnormalities can be present in low-grade tumors, suggesting that they are early events in the process of oligodendroglial cellular transformation [99]. Other studies have demonstrated that genes, such as the tumor suppressor, *AJAP1*, which are expressed at lower levels in oligodendrogliomas relative to normal brain tissues and associated with decreased survival, are often hypermethylated [100]. Interestingly, a recent report also demonstrated that the p53-dependent anti-apoptotic modulator PDAM, which is frequently deregulated in oligodendroglial tumors and involved in mediating chemosensitivity of these tumors, is not only subject to epigenetic regulation but also that it may itself function as a ncRNA [101].

#### 3.2.2. Medulloblastoma

A recent analysis revealed that mutations in epigenetic factors promote the pathogenesis of medullublastomas including, specifically, the histone lysine methyltransferases, *MLL2* and *MLL3*; histone lysine demethylase, *KDM6B*; and SWI/SNF family members, *SMARCA4* and *ARID1A* [102]. A complementary study showed that amplifications and homozygous deletions in genes responsible for modulating levels of histone lysine methylation, particularly H3K9, are involved in pathogenesis of medulloblastoma. These include the histone lysine methyltransferases, *EHMT1* and *SMYD4*; histone lysine demethylases, *JMJD2C* and *JMJD2B*; histone lysine acetyltransferase, *MYST3*; and PcG factors, *L3MBTL2*, *L3MBTL3*, and *SCML2* [103]. REST is another important epigenetic factor often overexpressed in medulloblastomas and implicated in the formation of tumors in the mouse cerebellum, when it is overexpressed along with c-Myc [104-107]. REST may be associated with the pathogenesis of these tumors because it modulates NSC differentiation, is involved in the Wnt and Sonic hedgehog (Shh) developmental pathways, controls the expression and functions of epigenetic regulators, including many of those listed above, and is integrated into ncRNA regulatory networks [108].

A number of studies have identified miRNAs that are deregulated in medulloblastomas, including those that are upregulated and downregulated [109-111]. Particular miRNAs may be linked to molecular pathways previously implicated in the development of these tumors. For example, mature miRNAs derived from the *miR-17-92* miRNA polycistronic cluster, which have previously been identified as oncomirs, are the most highly upregulated miRNAs in medulloblastomas—specifically the subset of these tumors associated with constitutive activation of Shh signaling [109,110]. Moreover, particular miRNA signatures can be combined with gene expression and copy number variation data to identify subtypes of medulloblastomas that have meaningful differences in clinical outcomes, such as higher and lower rates of event-free and overall survival [111].

Like GBMs, medulloblastomas can also participate in intercellular epigenetic signaling through the release of exosomes containing high levels of retrotransposon-associated DNA and RNA sequences including *LINE1*, *Alu* and *HERV-K* retrotransposon elements [97].

#### 3.2.3. Neuroblastoma

Studies performed utilizing neuroblastomas and related cell lines have demonstrated the presence of distinct profiles of DNA methylation at particular genomic loci and suggested that these profiles can distinguish between molecular subtypes of tumors associated with different clinical outcomes. For example, a recent DNA methylation analysis of 33,485 discrete loci in neuroblastic tumors and associated copy number analysis identified extensive epigenetic and large-scale epigenomic alterations (preferentially localized to telomeric regions) correlated with patient survival [112]. A range of other studies have similarly identified genes, which are methylated and silenced in neuroblastoma in various patterns, including but not limited to the tumor suppressor genes, *RASSF1A* and *ZMYND10* (a.k.a., *BLU*); the angiogenesis inhibitor, *THBS1* [113]; the anti-apoptotic decoy receptors, *DcR1* and *DcR2* [114]; the extrinsic pathway associated caspase, *CASP8* [115,116], various Shh pathway genes [117]; and other factors [118-120]. Hypermethylation of specific genes has been associated with certain clinical outcomes, such as relapse susceptibility (*i.e.*, *CASP8*) and overall survival (*i.e.*, *TMS1* and *APAF1*) [121]. By contrast, lower levels of methylation associated with particular genes (*i.e.*, *SCNN1A, PRKCDBP* and *KRT19*) are linked with more favorable outcomes [119]. Furthermore, while there can be a significant correlation between the methylation of certain genes (*i.e.*, *CASP8*) and previously described predictive factors for neuroblastoma (*i.e.*, *MYCN* amplification status) [122]; distinct methylation signatures, such as the so-called CpG island methylator phenotype (CIMP), can independently be associated with clinical prognosis regardless of these other factors [123-125]. Additionally, an initial analysis has also demonstrated the presence of differential profiles of histone modifications at the loci of some differentially methylated genes (*i.e.*, *THBS1*) in neuroblastomas, highlighting the complexity of epigenetic processes that may be involved in these tumors.

Specific epigenetic regulatory factors may, themselves, be deregulated in neuroblastomas. For example, increased expression levels [126] and changes in the alternative splicing patterns [127,128] of REST are found in neuroblastomas. Neuroblastomas also exhibit alterations in the expression of various miRNAs including, for example, those that modulate molecular pathways involved in apoptosis, cell survival and proliferation [129-131]. One interesting study demonstrated that neuroblastomas have increased levels of *miR-17-5p*, which inhibits p21 and BCL2L11 (a.k.a., BIM) expression, and that p21 downregulation is significant in tumors with *MYCN* amplification and a poor clinical outcome [132]. This study also showed that treatment of *MYCN*-amplified and therapy-resistant neuroblastomas with an oligonucleotide designed to block the action of *miR-17-5p*, a *miR-17-5p* antagomir, abolishes the p21 and BIM upregulation, inhibits cell cycle progression and promotes apoptosis [132]. Another study found that *miR-9*, a miRNA with tumor suppressor activity, is downregulated in 50% of neuroblastomas [130]. Furthermore, increased levels of REST have been implicated in downregulating *miR-9* expression in neuroblastoma cells [131].

#### 3.2.4. Other Nervous System Tumors

An increasing number of studies are revealing that other primary nervous system tumors are similarly associated with epigenetic deregulation or mutations in epigenetic regulatory factors. One example is that of ependymomas, which exhibit abnormal profiles of DNA methylation at various genomic loci including *RASSF1A* [133,134]; *CDKN2A/B* and *p14 ARF* [135]; and *MGMT*, *TIMP3*, *THBS1* and *TP73* [136]. In contrast to these candidate gene studies, a recent analysis of genome-wide patterns of DNA methylation in ependymomas revealed both gain and loss of methylation associated with a range of genomic loci, including specifically *Alu* repeat elements and their flanking sequences [137]. Further, hypomethylation of *Alu* elements was characteristic of aggressive primary tumors and even more so for recurrent tumor subtypes, suggesting that *Alu* methylation status may be a valuable prognostic marker [137]. Another example, rhabdoid tumors are largely caused by deletions or mutations of *SMARCB1* (a.k.a., *INI1/SNF5/BAF47*), a component of the chromatin remodeling SWI/SNF complex, which functions as a tumor suppressor by modulating the cell cycle and cooperating with p53 to prevent transformation [138].

## 4. The Cancer Stem Cell Hypothesis vis-à-vis Epigenetics

### 4.1. Cancer Stem Cells

In contrast to the canonical stochastic theory that attributes the molecular pathogenesis of cancer to clonal evolution, the hierarchical CSC hypothesis suggests that tumor maintenance, progression, treatment resistance, and recurrence are largely driven by cells with stem cell-like characteristics, including the capacity to undergo self-renewal and differentiate into the potentially heterogeneous subpopulations of cells present within a particular tumor. Studies on acute myeloid leukemia supplied preliminary data supporting the CSC hypothesis [139]. Subsequent studies have provided evidence for the existence of CSCs in a broad range of solid tumors including those that manifest in the CNS, such as gliomas, medulloblastomas, neuroblastomas, and ependymomas [140,141]. Cells derived from nervous system tumors are operationally defined as CSCs if they express certain cell surface markers (*i.e.*, CD133, CD15/SSEA-1, L1CAM, A2B5, or integrin a6) and exhibit particular functional properties (*i.e.*, the ability to initiate and propagate tumors in various assays). Our understanding of these so-called CSCs, their *in situ* interactions with the tumor microenvironment, and corresponding methodologies used to identify and study them *ex vivo* are still evolving. Furthermore, the relevance of the CSC model to specific cancers remains the subject of ongoing controversies [142], though stochastic and hierarchical models are not mutually exclusive [143,144].

### 4.2. Epigenetic Deregulation in Tumor Initiating and Propagating Cells Derived from Nervous System Tumors

Preliminary studies of tumor initiating and propagating cells, if not CSCs, derived from nervous system tumors have demonstrated that these cells are epigenetically deregulated. In fact, the PcG protein, BMI1, is highly enriched in GBM-derived CD133+ cells and is required for sustaining stem cell self-renewal. Further, examining GBMs and associated cell lines reveals that the *CD133* gene, itself, is subject to regulation by DNA methylation, though, conspicuously, this only seems to be the case in the cancer state [145]. The methylation status of the *CD133* gene promoter is generally inversely correlated with the expression of this cell surface marker in GBM-derived CD133+ and CD133- cell populations [145,146]. Also, the *CASP8* gene promoter is hypermethylated in GBM-derived CD133+ cells, leading to low levels of CASP8 expression in these cells [147]. Notably, this protein is required for mediating the sensitivity of the tumor to the tumor necrosis factor-related apoptosis-inducing ligand (TRAIL); and not surprisingly, these cells are resistant to TRAIL-induced cell death. The *BMP receptor 1B* gene, which typically inhibits proliferation and promotes NSC differentiation, is also epigenetically silenced in GBM-derived tumor initiating and propagating cells. It is subject to promoter hypermethylation and is also the target of the H3K27 histone methyltransferase, enhancer of zeste 2 (EZH2), a PcG protein [148]. In addition, comparative profiling of miRNAs reveals that many miRNAs are differentially expressed between GBM-derived CD133+ and CD133- cells [149]. Specifically, miRNAs that may have roles in promoting brain development and neural cellular differentiation, such as *miR-451*, *miR-486* and *miR-425* [149] and *miR-29b*, *miR-125a* and *miR-149* [150], are upregulated in CD133- cells. Moreover, the introduction of *miR-124* or *miR-137* inhibits proliferation and induces differentiation in GBM-derived (and oligodendroglioma-derived) tumor initiating and propagating cells [151]. Neuroblastoma- and medulloblastoma-derived tumor initiating and propagating cells are also subject to epigenetic regulation, though, these processes are less well characterized than those operating in GBM-derived tumor initiating and propagating cells. For example, the *CD133* gene is similarly subject to regulation by DNA methylation in neuroblastoma cell lines [152]. And, in medulloblastoma, the introduction of *miR-199b-5p* depletes the CD133+ population of cells and reduces the expression of genes linked to the stem cell state, such as HES1 [153].

Given the recognition of the increasingly roles of epigenetic mechanisms in mediating all aspects of neural cellular development, these observations suggest that epigenetic deregulation is a critical factor for determining tumor initiating and propagating cell functions.

### 4.3. Epigenetic Mechanisms and the Cancer Stem Cell State

By definition, CSCs have the ability to undergo self-renewal and to differentiate into potentially heterogeneous subpopulations of cells that comprise an individual tumor. CSCs are, thus, responsible for maintaining and propagating tumors. However, our understanding of the processes by which CSCs are generated and maintained in nervous system (and other) tumors that conform to the CSC model remains limited. Many studies have focused on identifying the cells of origin—cells that acquire genetic or other lesions and are truly responsible for initiating nervous system tumors *in situ* [154]. Indeed, it has been shown that GBMs, medulloblastomas, neuroblastomas, and other nervous system tumors can arise from cells of origin including neural stem and progenitor cells that escape from normal proliferation and differentiation programs and even from more mature cell types that undergo de-differentiation. For example, both NSCs or cerebellar granule neuronal precursor cells can form medulloblastomas [155], and mature macroglial cells, such as astrocytes, can form gliomas. These cells of origin are distinct from CSCs, but they may be closely related to and are either directly of indirectly responsible for generating CSCs [154]. Because epigenetic mechanisms are implicated in mediating neural cell identity and function and also because they are deregulated in cancer generally and in populations of tumor initiating and propagating cells derived from nervous system tumors, epigenetic deregulation is likely to be a very important pathogenic mechanism in the cancer cell of origin and in the establishment and maintenance of the CSC state [156].

Indeed, emerging evidence has demonstrated that epigenetic mechanisms play roles in mediating all of the major acquired abilities that are the hallmarks of cancer and the characteristics that enable them (recently reviewed by Hanahan and Weinberg [157]). These properties include the capacity to sustain proliferative signaling, evade growth suppressors, resist cell death, achieve replicative immortality, induce angiogenesis, activate invasion and metastasis, reprogram energy metabolism and evade immune destruction and are underpinned by genome instability and inflammation and through bidirectional interactions with the tumor microenvironment. Here we provide leading examples of how diverse epigenetic mechanisms are involved in the molecular circuitry underlying all of these pathological processes. Proliferative signaling pathways include those that are mediated by growth factors and related developmental cues. REST, an epigenetic regulatory factor deregulated in nervous system tumors, modulates Shh signaling [158], and in turn, the activity of REST is modulated by the Wnt signaling pathway [159,160]. Growth suppressors, such as the pRb and p53 tumor suppressors, themselves, participate in epigenetic pathways. For example, the mechanism of action of pRb includes modulation of local and more global chromatin environments by recruitment of enzymes that modify histones, remodel nucleosomes, and promote the formation of heterochromatin [161]. Similarly, p53 binds to its response element, where it recruits coregulatory factors such as histone modifying enzymes, chromatin remodeling factors, and subunits of the mediator complex. This stimulus-specific p53 activity is also important for sensing cell stress and promoting apoptosis. Replicative immortality is mediated by telomeres, which are, by definition, chromosomal structures. The organization of telomeric chromatin influences telomere maintenance, and telomere dysfunction affects telomeric and subtelomeric chromatin formation and activates alternative telomere maintenance mechanisms [162]. Further, treatment of nervous system tumor cells with HDAC inhibitors has been shown to have antiproliferative and proapoptotic effects, mediated by alterations in chromatin at the telomerase reverse transcriptase (TERT) promoter [163]. Moreover, telomeres are transcribed into ncRNAs, telomere repeat-containing RNA (TERRA), that may have roles in modulating telomeric heterochromatin formation, the accessibility of telomeres to DNA repair mechanisms, and the activity of telomerase [164]. Angiogenesis is regulated by the actions of various pro- and anti-angiogenic factors including many, such as VEGF, with expression levels that are modulated by DNA methylation, histone modifications, and microRNAs [165]. In turn, VEGF influences expression of the PcG protein, EZH2, in the tumor vasculature. EZH2 promotes tumor angiogenesis by repressing the anti-angiogenic factor, Vash1 [166]. Invasion and metastasis are also mediated by epigenetic factors such as ncRNAs. The expression of certain miRNAs can correlate positively or negatively with the invasive and metastatic phenotype of cancer. For example, *miR-10b* is a miRNA that promotes invasion and metastasis in gliomas [167]. In contrast, *miR-124a* is generally downregulated in GBMs, and ectopic expression of *miR-124a* in GBM cells inhibits tumor invasion and migration [168]. In addition, expression of certain lncRNAs is also correlated with the phenotype of particular cancers. Higher levels of HOTAIR, a lincRNA transcribed from the HOX locus, are implicated in promoting metastasis [36] and recurrence [169] and indicate a poor clinical prognosis for particular cancers. Many aspects of energy metabolism are also modulated by epigenetic factors. In fact, HDACs play a pivotal role in glucose homeostasis by regulating Forkhead box O transcription factors, which have critical roles in modulating metabolism [170,171]. The immune response and its evasion by cancer cells are also mediated by epigenetic factors, including HDACs. For example, HDACs are implicated in transcriptional regulation of both pro- and anti-inflammatory cytokines, in immune cell differentiation, in immunological signaling pathways (*i.e.*, STAT and NF-κB) and inflammatory responses, in forming the antigen-presenting cell/T-cell immunological synapse, and specifically in modulating the immune response to cancer [172].

Tumor microenvironments (or niches) include the extracellular matrix (ECM) and various stromal, endothelial, and other cell types that create the local milieu permitting tumor initiation and subsequently supporting tumor maintenance and progression. The bidirectional communication that exists between CSCs and tumor microenvironments located in perivascular and, putatively, in hypoxic and invasive edge regions is modulated, at least in part, by epigenetic factors. For example, GBM CSCs secrete VEGF that promotes endothelial cell growth [173], and we have already noted how VEGF signaling is under epigenetic control. In addition, Notch signaling maintains CSCs [174], and many epigenetic factors such as histone modifying enzymes and corepressor complexes and associated epigenetic marks are important for regulating Notch target gene expression [175]. Furthermore, it is likely that many other bidirectional communications are mediated by epigenetic factors. Indeed, integrins and ECM interactions are important for CSC maintenance [176], and emerging evidence suggests an epigenetic level of control for these factors, mediated particularly by REST and CoREST [16-18,177]. Diffusible cerebrospinal fluid-borne signals that impact NSC proliferation, including specifically those derived from patients with GBMs, are partly dependent on insulin-like growth factor 2 (IGF2) levels [178], and IGF2 is regulated by intricate epigenetic mechanisms including imprinting and lncRNAs [179,180]. The release of exosomes containing regulatory and functional ncRNAs, including those derived from transposable elements, also represents an intriguing mechanism for bidirectional intercellular communication in the tumor microenvironment, and more general evidence already exists for such neural-endothelial cell communication promoting an angiogenic phenotype [96,181].

## 5. Epigenetic Medicine

Fundamental biological questions have yet to be answered regarding the precise roles played by epigenetic deregulation in cancer pathogenesis, generally, and in the emergence, maintenance and evolution of CSCs, in particular. Nevertheless, molecular diagnostics evaluating epigenetic factors and processes and complementary therapeutic agents targeting epigenetic factors are very actively being developed for a variety of tumors and have even been approved by the FDA for selected indications. In fact, *MGMT* promoter methylation and CIMP are clearly clinically relevant biomarkers. In addition, agents currently used to treat nervous system tumors, such temozolomide and carmustine, exert their effects, at least in part, by impacting epigenetic states including DNA methylation status, chromatin structure and nuclear organization [182]. Thus, the broad range of observations that we have highlighted here provides strong evidence supporting the vigorous advancement of existing and novel epigenetic strategies in concert with other approaches for diagnosing and treating nervous system tumors, including those focusing on the elimination of CSCs.

Many agents that can modulate DNA methylation have been identified. The best-characterized DNMT enzyme inhibitors include 5-azacytidine, 5-aza-2-deoxycytidine, and zebularine. Some commonly used drugs, such as hydralazine, procainamide and valproic acid (VPA), can also influence DNA methylation profiles [183]. In addition, a number of drugs that can modulate histone-modifying enzyme activity are also available. The best-described agents are the HDAC inhibitors, which include trichostatin A, suberoylanilide hydroxamic acid, sodium butyrate, sodium 4-phenylbutyrate, romidepsin and VPA. Each of these drugs differentially inhibits the activity of the 5 major classes of HDAC enzymes and of individual HDAC isotypes. In pre-clinical studies, various HDAC inhibitors, alone or in combination with other therapies, have shown significant anti-cancer effects through a broad range of cellular mechanisms, including but not limited to the modulation of oncogene and tumor suppressor gene expression, cell cycle, intrinsic and extrinsic apoptotic pathways, autophagy, reactive oxygen species generation, angiogenesis, inflammation, immunosurveillance, DNA repair, and radiosensitization. Correspondingly, the most notable clinical trials that have been performed, and those that are currently underway, utilizing epigenetic therapeutic agents to treat nervous system tumors—primarily gliomas—have focused on studying the effects of HDAC inhibitors alone or in combination with other modalities, such as chemotherapies, biologic therapies, or radiation therapies (reviewed in [184]). Despite important advances, however, these first-generation epigenetic drugs are non-specific and often associated with off-target effects and significant toxicity, thus more selective agents that affect these pathways are being developed. Moreover, small molecules and other agents targeting a more diverse range of histone- and chromatin-modifying factors are being identified or designed at a rapid pace. For example, compounds, such as BIX-01294 and chaetocin, can inhibit G9a and SUV39H1 histone methyltransferase activity [185,186]; certain monoamine oxidase inhibitors, such as trans-2-phenylcyclopropylamine, can inhibit LSD1 histone demethylase activity [187-189]; agents, such as the S-adenosylhomocysteine hydrolase inhibitor 3-deazaneplanocin and radicicol, can modulate the levels and functions of PcG and TrxG proteins, respectively [190,191]; and, compounds, such 2-aminothiazole derivatives, can inhibit REST activity [192-194].

Our evolving understanding of ncRNA biology suggests that interrogating these factors and targeting them directly, or indirectly via their biogenesis and effector pathways, represent important and potentially more sensitive and specific strategies for diagnosing and treating nervous system tumors. For example, sequencing genomic DNA can be used to identify variations in regions that give rise to ncRNAs and in regions of cancer-associated genes to identify pathogenic variability in ncRNA-mRNA interactions that may result in altered target gene regulation. Further, the existence of various classes of ncRNAs with highly pathological cell- and tissue-selective expression profiles implies that these factors may be more effective molecular biomarkers and mechanistic targets for identifying and modifying cancer pathogenesis compared to existing approaches. Indeed, molecular diagnostics that measure the levels of certain classes of ncRNAs, such as miRNAs, are already in relatively advanced stages of development, both for cancer and for other disorders [195-200]. Other ncRNAs may similarly be useful as biomarkers for risk stratification, screening, prognostication, customization of therapies, and monitoring treatment responses and disease recurrence [201,202]. Intriguingly, ncRNA levels in peripheral tissues may even provide signatures of central disease activity, as significant correlations are present between ncRNA expression profiles in brain and those in other tissues, such as blood, in certain disease processes [203,204]. Moreover, exosomes containing various ncRNAs circulate in the blood of patients with nervous system tumors and may play roles in promoting malignant transformation and dissemination and in suppressing immune responses [96,205]. In addition, small molecule, oligonucleotide and related treatment approaches are also being developed with the potential to disrupt and inhibit, or to replace and promote, ncRNA expression and function [206,207]. While many of these emerging epigenetic therapeutic approaches are very promising, significant technical barriers also exist for designing and delivering specific agents to the brain. Can these molecules be engineered to very selectively modulate a particular epigenetic regulatory factor or pathway and optimized to promote particular gene expression programs (*i.e.*, those associated with survival in healthy cells but cell death in cancer cells and in CSCs, specifically)? Can these agents be delivered successfully to the tumor and to CSCs without off-target effects in the brain, vascular, immune, and other systems to minimize toxicities, perhaps through emerging neuro-interventional approaches?

## 6. Conclusions

It is clear that understanding nervous system tumors in general and the origins and behavior of CSCs in particular requires a deeper appreciation for epigenetic mechanisms that modulate neural cell identity, including their interplay with genetic and other cell-intrinsic factors and cell-extrinsic and cell-cell cues, such as those derived from the microenvironment. Efforts are underway to collect and analyze complex genomic, transcriptomic, and epigenomic data from nervous system tumors and to correlate these molecular signatures with clinical features and outcomes [208]. These studies will hopefully lead to earlier detection and better risk stratification and guide the development of more effective and even preventive therapies for nervous system tumors.

## Figures and Tables

**Table 1.** Principal epigenetic regulatory mechanisms and associated examples of factors implicated in mediating these processes.

**Epigenetic mechanism**	**Epigenetic factors**
DNA (de)methylation and hydroxymethylation	DNA methyltransferase enzymes Methyl-CpG-binding domain proteins DNA excision repair proteins Cytidine deaminase enzymes Gadd45 proteins Ten-Eleven Translocation enzymes
Histone and chromatin modifications	Histone modifying enzymes (e.g., histone [de]acetylase and [de]methylase) Polycomb group proteins Trithorax group proteins REST and CoREST epigenetic regulatory complexes
Non-coding RNAs (ncRNAs)	Short-interfering RNAs MicroRNAs PIWI-interacting RNAs Small nucleolar RNAs Long ncRNAs Long intergenic ncRNAs
RNA editing	Adenosine deaminase that act on RNA enzymes Apolipoprotein B editing catalytic subunit enzymes
Nuclear organization	Nuclear domains (e.g., nuclear speckles) Nuclear lamina (e.g., lamin genes)

## Abbreviations

5hmC: 5-hydroxymethylcytosine
5mC: 5-methylcytosine
ac: acetylation
ADAR: adenosine deaminase that act on RNA
APOBEC: apolipoprotein B editing catalytic subunit
CIMP: CpG island methylator phenotype
CNS: central nervous system
CSC: cancer stem cell
DNMT: DNA methyltransferase
ECM: extracellular matrix
ESC: embryonic stem cell
EZH2: enhancer of zeste 2
GBM: glioblastoma multiforme
H: histone
HAT: histone acetylase
HDAC: histone deacetylase
K: lysine
lincRNA: long intergenic ncRNA
lncRNA: long ncRNA
MBD: methyl-CpG-binding domain protein
me: methylation
MGMT: O (6)-methylguanine-DNA methyltransferase
miRNA: microRNA
mRNA: messenger RNA
ncRNA: non-coding RNA
NPC: neural progenitor cell
NSC: neural stem cell
OL: oligodendrocyte
PcG: Polycomb group
Shh: sonic hedgehog
siRNA: short interfering RNA
snoRNA: small nucleolar RNA
TERRA: telomere repeat-containing RNA
TERT: telomerase reverse transcriptase
TET: Ten-Eleven Translocation
TRAIL: tumor necrosis factor-related apoptosis-inducing ligand
TrxG: Trithorax group
UTR: untranslated region
VPA: valproic acid
XCI: X chromosome inactivation
